# Eukaryote-Made Thermostable DNA Polymerase Enables Rapid PCR-Based Detection of Mycoplasma, Ureaplasma and Other Bacteria in the Amniotic Fluid of Preterm Labor Cases

**DOI:** 10.1371/journal.pone.0129032

**Published:** 2015-06-04

**Authors:** Tomohiro Ueno, Hideki Niimi, Noriko Yoneda, Satoshi Yoneda, Masashi Mori, Homare Tabata, Hiroshi Minami, Shigeru Saito, Isao Kitajima

**Affiliations:** 1 Clinical Laboratory Center, Toyama University Hospital, Toyama, 930–0194, Japan; 2 Department of Obstetrics & Gynecology, Toyama University Hospital, Toyama, 930–0194, Japan; 3 Research Institute for Bioresources and Biotechnology, Ishikawa Prefectural University, Ishikawa, 921–8836, Japan; 4 Life Science Center, Hokkaido Mitsui Chemicals, Inc., Hokkaido, 073–0138, Japan; Seattle Childrens Hospital, UNITED STATES

## Abstract

**Background:**

Intra-amniotic infection has long been recognized as the leading cause of preterm delivery. Microbial culture is the gold standard for the detection of intra-amniotic infection, but several days are required, and many bacterial species in the amniotic fluid are difficult to cultivate.

**Methods:**

We developed a novel nested-PCR-based assay for detecting *Mycoplasma*, *Ureaplasma*, other bacteria and fungi in amniotic fluid samples within three hours of sample collection. To detect prokaryotes, eukaryote-made thermostable DNA polymerase, which is free from bacterial DNA contamination, is used in combination with bacterial universal primers. In contrast, to detect eukaryotes, conventional bacterially-made thermostable DNA polymerase is used in combination with fungal universal primers. To assess the validity of the PCR assay, we compared the PCR and conventional culture results using 300 amniotic fluid samples.

**Results:**

Based on the detection level (positive and negative), 93.3% (280/300) of *Mycoplasma*, 94.3% (283/300) of *Ureaplasma*, 89.3% (268/300) of other bacteria and 99.7% (299/300) of fungi matched the culture results. Meanwhile, concerning the detection of bacteria other than *Mycoplasma* and *Ureaplasma*, 228 samples were negative according to the PCR method, 98.2% (224/228) of which were also negative based on the culture method. Employing the devised primer sets, mixed amniotic fluid infections of *Mycoplasma*, *Ureaplasma* and/or other bacteria could be clearly distinguished. In addition, we also attempted to compare the relative abundance in 28 amniotic fluid samples with mixed infection, and judged dominance by comparing the Ct values of quantitative real-time PCR.

**Conclusions:**

We developed a novel PCR assay for the rapid detection of *Mycoplasma*, *Ureaplasma*, other bacteria and fungi in amniotic fluid samples. This assay can also be applied to accurately diagnose the absence of bacteria in samples. We believe that this assay will positively contribute to the treatment of intra-amniotic infection and the prevention of preterm delivery.

## Introduction

Intra-amniotic infection has long been recognized as the leading cause of preterm delivery [1–3], and preterm birth is a major cause of neonatal mortality worldwide [4]. Bacterial invasion in the amniotic fluid induces an inflammatory response. This has been shown to be a crucial factor for the onset of spontaneous abortion, chorioamnionitis, preterm premature rupture of the fetal membranes and preterm labor (PTL) [5–8]. Furthermore, infection of the infant *in utero* leads to an increased risk of perinatal morbidity, including pneumonia, bacteremia or meningitis [9, 10]. A variety of microorganisms have been cultivated from amniotic fluid in pregnancies complicated by preterm birth [1, 11, 12], but in particular, *Mycoplasma* and *Ureaplasma* species are the most frequently isolated pathogens in cases with intra-amniotic infection [6, 13–15].

Microbial culture of amniotic fluid is recognized as the “gold standard” for the detection of intra-amniotic infection, but several days are usually required to detect bacteria and/or fungi in amniotic fluid samples, and a high percentage of bacterial species in the amniotic fluid are difficult to cultivate [16, 17]. Polymerase chain reaction (PCR) has become an important tool for the rapid, sensitive and specific detection of bacteria without the need to culture them [18]. PCR-based methods can detect pathogens, including difficult-to-cultivate bacteria. In fact, the identification of *Mycoplasma* and *Ureaplasma* species by PCR improved the detection rate of these microorganisms in comparison to culture-dependent methods [19–23]. A quantitative real-time PCR (qPCR) assay for the detection of bacteria is also important to determine the load of pathogenic microorganisms. The quantification of microorganisms by ordinary culture methods in clinical samples is not as accurate as qPCR.

Concerning bacterial universal PCRs, the main problem is the presence of contaminating bacterial DNA in commercial preparations of recombinant thermostable DNA polymerases as a result of its manufacture and incomplete purification [24–27]. To solve the problem, we developed the eukaryote-made thermostable DNA polymerase, which is free from bacterial DNA contamination [28]. Using eukaryote-made thermostable DNA polymerase, the sensitive and reliable detection of bacteria becomes feasible in practice for various fields.

In this manuscript, using the eukaryote-made thermostable DNA polymerase, we report the development of a novel nested-PCR-based assay for the rapid detection of *Mycoplasma*, *Ureaplasma*, other bacteria (bacteria other than *Mycoplasma* and *Ureaplasma*) and fungi in amniotic fluid samples in order to support the successful treatment of patients with PTL. This method can also be used to rapidly diagnose the absence of bacteria in amniotic fluid samples. Moreover, to judge dominance, we report a trial of qPCR for comparing the relative abundance of *Mycoplasma*, *Ureaplasma* and/or other bacteria in amniotic fluid with mixed infection.

## Materials and Methods

### Study participants and clinical sample collection

A total of 5–10 mL amniotic fluid samples were collected from 205 pregnant women in preterm labor (age range:18–43 years, gestational age: 22–40 weeks) by transabdominal (62 cases) or transvaginal (35 cases) amniocentesis, or at the time of caesarean section (92 cases) or vaginal delivery (16 cases) at Toyama University Hospital. In addition, a total of 5–10 mL amniotic fluid samples were collected from 95 pregnant women without preterm labor (age range: 21–45 years, gestational age: 35–41 weeks) at the time of cesarean section at Toyama University Hospital. In cases of vaginal delivery, transvaginal amniocentesis was performed when crowning occurred. In cases of caesarean section, amniocentesis was performed before cutting the fetal membrane. Written informed consent was obtained from the patients for the collection and use of the clinical samples. This study was conducted with the approval of the Ethics Committee on Genomic Research of the University of Toyama.

### DNA extraction from amniotic fluid samples

One mL of amniotic fluid or, in the case of extraction control, 1 mL of distilled water (water deionized and sterilized for molecular biology, NACALAI TESQUE, INC. Kyoto) was centrifuged at 20,000×g for 20 minutes to spin down the microorganisms, and 950 μL of the supernatant fraction was carefully removed in order to not lose the pellet, leaving the pellet with 50 μL of supernatant. DNA was isolated from the resulting pellet using a DNA extraction kit (High Pure PCR Template Preparation Kit, Roche Applied Science, Germany) in accordance with the supplier’s instructions. Finally, the bacterial DNA was eluted with 100 μL of elution buffer.

### Nested PCR assays for detecting *Mycoplasma*, *Ureaplasma* and other bacteria

The following is a nested PCR procedure (first PCR: 30 cycles ➞ dilute 500-fold ➞ second, nested PCR: 30 cycles). The LightCycler Nano (Roche Applied Science) was used for the amplification and real-time detection of the target DNA. We used 1.5 mL PCR-clean Eppendorf tubes that were RNase- and DNase-Free (Eppendorf, Germany), and 0.1 mL PCR Tubes (Roche Diagnostics). All oligonucleotide primers were designed using a multiple alignment software program (Clustal X) comparing more than 200 kinds of bacterial 16S rRNA sequences, and were synthesized by Life Technologies Japan, Ltd. (Tokyo, Japan). The primer information is shown in Table 1.

**Table 1:** The PCR primers and amplicon sizes in base pairs.

Primer pair name	Primer sequence (5’➞3’)	Primer position	Tm degree* (°C)	Amplicon size (bp)
Bacterial Universal Primer for 1^st^ PCR	F- AGAGTTTGATCATGGCTCAG	8–27*^1^	60.7	1379
	R- CCGGGAACGTATTCACC	1369–1385*^1^	62.7	
Bacterial Universal Primer for 2^nd^ PCR	F- AGCAGCCGCGGTAATA	519–534*^1^	61.8	287
	R- GGACTACCAGGGTATCTAATCCT	783–805*^1^	61.4	
NotMycoUrea Bacterial Universal Primer	F- TGGTTTAATTCGATGCAACGC	951–971*^1^	67.9	120
	R- GAGCTGACGACAGCCAT	1054–1070*^1^	61.4	
Mycoplasma Specific Primer	F 1- GACGTGTAGCTATGCTGAGA	281–300*^2^	59.5	169 or 173
	F 2- GTTTAGCCGGGTCGAG	277–292*^3^	60.6	
(F1, F2, and R1, R2 are mixed in one tube)	R 1- TTCTTCCCAAATAAAAGAACTTT	431–453*^2^	60.1	
	R 2- TTCTTCCCTTATAACAGCACTTT	423–445*^3^	61.0	
Ureaplasma Specific Primer	F- TAACATCAATATCGCATGAGAAG	179–201*^4^	61.8	128
	R- CAGTACAGCTACGCGTCATT	287–306*^4^	61.8	
Fungal Universal Primer	F- CTTTCGATGGTAGGATAGTGG	210–230*^5^	61.3	615
	R- GCTTTCGCAGTAGTTAGTCTTC	802–823*^5^	60.2	

* Melting temperatures of the primers were calculated using the formula based on the nearest neighbor thermodynamic theory.

The target genes of the primer position are as follows:

*^1^: *Escherichia coli* 16S ribosomal RNA (Accession No. J01859)

*^2^: *Mycoplasma genitalium* 16S ribosomal RNA (Accession No. NR_074611)

*^3^: *Mycoplasma hominis* 16S ribosomal RNA (Accession No. NR_074603)

*^4^: *Ureaplasma parvum* 16S ribosomal RNA (Accession No. NR_074762)

*^5^: *Candida albicans* 18S ribosomal RNA (Accession No. AF114470)

During the first PCR procedure, all reactions were performed in one tube. The PCR reaction mixture (20 μL) contained 2 μL of DNA template or, as a positive control, 2 μL (8.0 ng/μL) of DNA extracted from *Escherichia coli* (ATCC 25922) or, as a negative control for the PCR step, 2μL of distilled water (water deionized and sterilized for molecular biology, NAKALAI TESQUE, INC.) in 200 μM of each dNTP (CleanAmp Hot Start dNTP Mix, SIGMA-ALDORICH, USA) filtered using an Amicon Ultra 50K centrifugal filter (Merck Millipore, Germany), 50 mM KCl, 2.25 mM MgCl_2_, 10 mM Tris-HCl (pH 8.3), 0.3 μM each of Bacterial Universal Primer for 1^st^ PCR, 1×EvaGreen (Biotium Inc. CA, USA), and 1.0 unit (0.5 μL) of eukaryote-made thermostable DNA polymerase supplemented with stock buffer solution. The generation of eukaryote-made thermostable DNA polymerase using *Saccharomyces cerevisiae* has been described previously [28].

The sample was incubated for five minutes at 95°C to activate the Hot Start dNTPs, then was denatured for 10 seconds at 95°C, annealed for 15 seconds at 57°C, extended for 30 seconds at 72°C and subjected to fluorescence acquisition for two seconds at 82°C for 30 cycles. The PCR product was diluted 500-fold with distilled water (water deionized and sterilized for molecular biology, NACALAI TESQUE, INC.) and then used as a template for the second (nested) PCR procedure. Even if no amplification curve was observed by the 30th cycle in the first PCR, the second (nested) PCR was still performed.

For the second (nested) PCR, all reactions were performed in four tubes for detecting bacteria, *Mycoplasma*, *Ureaplasma*, and bacteria other than *Mycoplasma* and *Ureaplasma*, respectively. The PCR reaction mixture (20 μL) contained 8 μL of DNA template of the diluted first PCR product in 200 μM of each dNTP (CleanAmp Hot Start dNTP Mix, SIGMA-ALDORICH) filtered using an Amicon Ultra 50K centrifugal filter (Merck Millipore), 50 mM KCl, 2.5 mM MgCl_2_, 10 mM Tris-HCl (pH 8.3), 0.25 μM each of each primer (Bacterial Universal Primer for 2^nd^ PCR, Mycoplasma Specific Primer, Ureaplasma Specific Primer, NotMycoUrea Bacterial Universal Primer), 1×EvaGreen (Biotium, Inc.) and 1.0 unit (0.5 μL) of eukaryote-made thermostable DNA polymerase supplemented with stock buffer solution. Each sample was incubated for five minutes at 95°C to activate the Hot Start dNTPs, then denatured for 10 seconds at 95°C, annealed for 15 seconds at 57°C, extended for 10 seconds at 72°C and subjected to fluorescence acquisition for two seconds at 82°C for 30 cycles. If no amplification curve was observed by the 30th cycle in the second (nested) PCR, we defined the sample as containing no bacteria. The presence of *Mycoplasma*, *Ureaplasma* and/or other bacteria were judged according to the real-time detection of target DNA. In addition, amplicons were further confirmed by agarose gel electrophoresis (2% agarose gel, ethidium bromide staining) or microchip electrophoresis (MCE-202 MultiNA, SHIMADZU, Japan).

A comparison of the relative abundance of *Mycoplasma*, *Ureaplasma* and/or other bacteria was subsequently performed, and a quantification cycle value was calculated using the LightCycler Nano software program.

### PCR assays for detecting fungi

To detect fungi, the LightCycler Nano (Roche Applied Science) and PCR-clean tubes were used as described above. During the PCR, the PCR reaction mixture (20 μL) contained 2 μL of DNA template or 2 μL (8.0 ng/μL) of DNA extracted from *Candida albicans* as a positive control, or distilled water (NAKALAI TESQUE, INC.) as a negative control in 50 mM KCl, 2.5 mM MgCl_2_, 10 mM Tris-HCl (pH 8.3), 200 μM of each deoxynucleoside triphosphate (dNTP), 0.25 μM each of Fungal universal primer, 1×EvaGreen (Biotium Inc.), and 2.0 units (0.4 μL) of conventional thermostable DNA polymerase (r-Taq: Toyobo, Osaka, Japan) supplemented with stock buffer solution. Each sample was incubated for three minutes at 95°C, then denatured for 10 seconds at 95°C, annealed for 15 seconds at 57°C, extended for 20 seconds at 72°C and subjected to fluorescence acquisition for two seconds at 82°C for 40 cycles.

### Culture-based detection of *Mycoplasma*, bacteria other than *Mycoplasma* and *Ureaplasma*, and fungi

The amniotic fluid samples were analyzed according to standard methods used by the Clinical Laboratory Center (certified ISO15189) at Toyama University Hospital. First, 1 mL amniotic fluid sample was centrifuged at 1,880×g for 15 min to spin down the microorganisms, and 800 μL of the supernatant fraction was carefully removed in order to not lose the pellet, leaving the pellet with 200 μL of supernatant. One drop of the resulting pellet with 200 μL of supernatant was placed on the appropriate agar media (PPLO agar for *Mycoplasma*, Brucella HK agar for anaerobic bacteria, blood agar, BTB agar and chocolate agar, respectively) and incubated aerobically or anaerobically until sufficient growth was present to proceed with testing (PPLO agar was incubated anaerobically at 35°C with 10% CO_2_ for up to 7 days, Brucella HK agar was incubated anaerobically at 35°C with 10% CO_2_ for up to 72 hours, blood agar and BTB agar was incubated aerobically at 35°C for up to 72 hours, and chocolate agar was incubated aerobically at 35°C with 5% CO_2_ for up to 72 hours). For all samples, the specific identification methods differed according to the organism, although they included the MicroScan WalkAway system (Siemens Healthcare Diagnostics, IL, USA), RapID ANA II (Thermo Fisher SCIENTICIC, UK) and various latex agglutination and biochemical spot tests.

### Culture-based detection of *Ureaplasma*

1 mL amniotic fluid sample was centrifuged at 1,880×g for 15 min to spin down the microorganisms, and 800 μL of the supernatant fraction was carefully removed in order to not lose the pellet, leaving the pellet with 200 μL of supernatant. The resulting pellet with 200 μL of supernatant was suspended in UMCHs medium: *Mycoplasma* broth base (Becton, Dickinson and Co., Baltimore, MD) 1.47% (wt/vol), 2.5% (wt/vol) yeast extract (Becton, Dickinson and Co.), 20% (vol/vol) horse serum (Biowhittaker, Walkersville, MD), 1.0% (vol/vol) supplement VX (Becton, Dickinson and Co.), 0.04% (wt/vol) urea, 0.001% (wt/vol) phenol red, 0.01% (wt/vol) L-cysteine hydrochloride, and 1000 U/mL penicillin G. After incubation at 35°C for up to 72 h, the color of the medium changed from yellow to red due to the hydrolysis of urea, and these color changes were regarded as indicating positivity for *Ureaplasma spp*. To confirm the presence of *Ureaplasma spp*., we also detected *Ureaplasma spp*. by colony formation.

## Results

### Workflow of the rapid detection method for *Mycoplasma*, *Ureaplasma*, other bacteria and fungi in amniotic fluid samples

**Fig 1:**
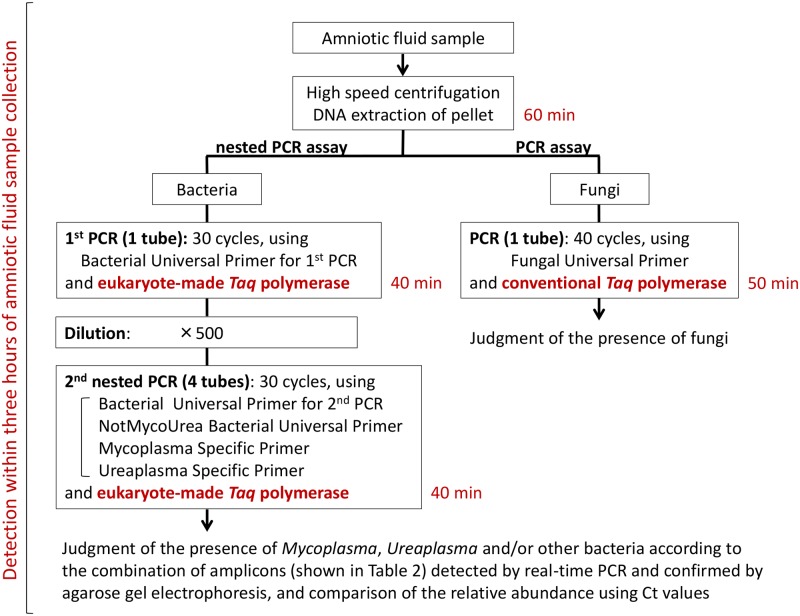
The workflow of the rapid detection method for *Mycoplasma*, *Ureaplasma*, other bacteria and fungi in amniotic fluid samples. Using this PCR-based method, pathogens can be detected within three hours of amniotic fluid sample collection. To prevent the occurrence of unreliable results in PCR-based assaying of amniotic samples for bacterial pathogens, eukaryote-made thermostable DNA polymerase (or *Taq* polymerase), which is free from bacterial DNA contamination, is used in combination with bacterial universal primers (along with two specific primers in the second, nested PCR). In contrast, for fungal pathogens, conventional bacterially-made thermostable DNA polymerase (or *Taq* polymerase), which is usually free from fungal DNA contamination, is used in combination with fungal universal primers.

Using this PCR-based method, pathogens can be detected within three hours of amniotic fluid sample collection (Fig 1). The workflow of the detection method is divided into two parts. One part is the detection of *Mycoplasma*, *Ureaplasma* and other bacteria, and the other part is the detection of fungi. To prevent the occurrence of unreliable results in PCR-based assays of amniotic fluid samples for both bacterial and fungal pathogens because of contamination by bacterial or fungal DNA, two kinds of thermostable DNA polymerase are used. That is, to detect prokaryotes such as *Mycoplasma*, *Ureaplasma* and other bacteria, eukaryote-made thermostable DNA polymerase, which is free from bacterial DNA contamination [28], is used in combination with bacterial universal primers. In contrast, to detect eukaryotes such as fungi, conventional bacterially made thermostable DNA polymerase, which is usually free from fungal DNA contamination, is used in combination with fungal universal primers. Consequently, promising detection of bacteria and fungi with a minimum contamination risk makes it possible to obtain more accurate diagnostic results, which can be useful for the management of preterm labor cases.

**Fig 2:**
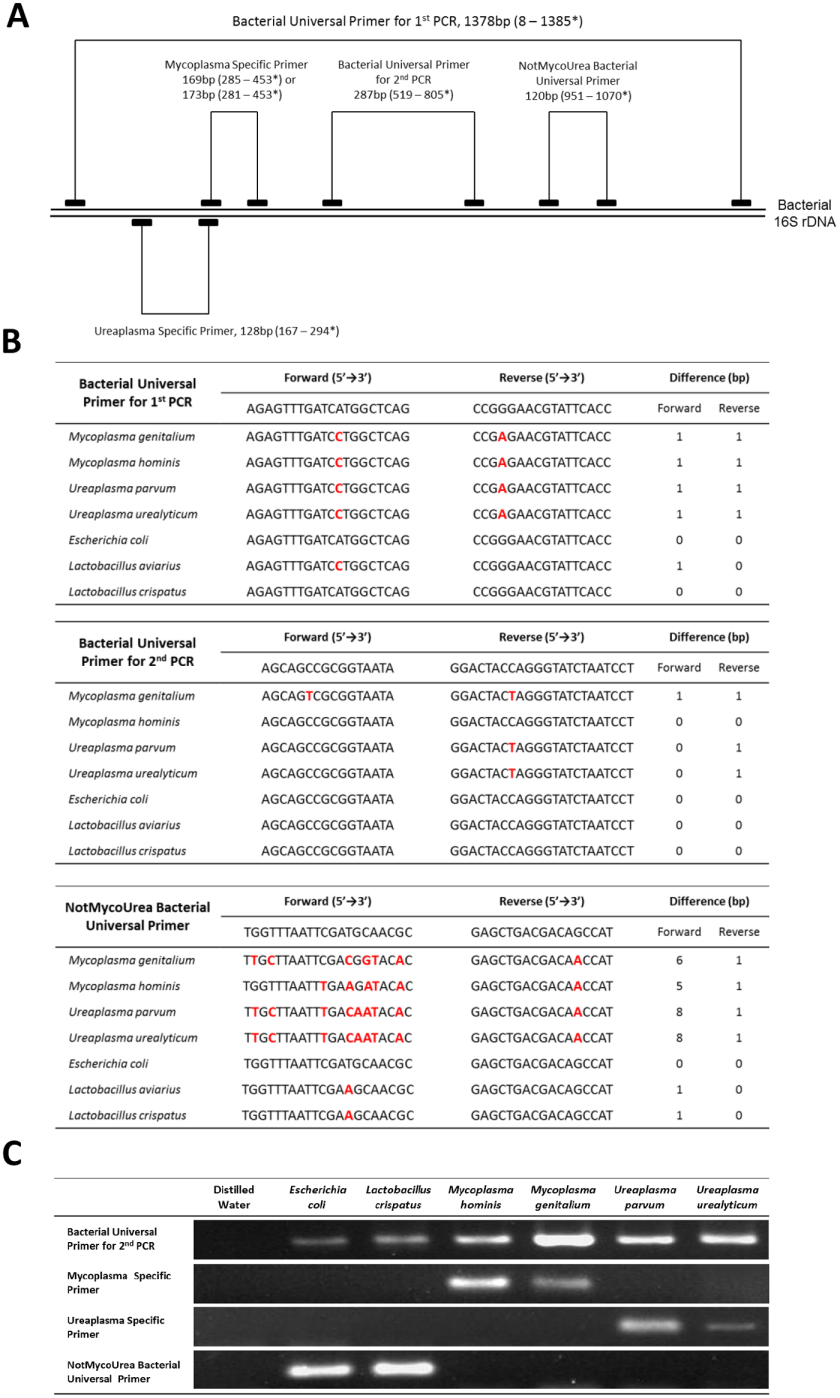
The strategy used for the primer design. (**A**) In an attempt to detect *Mycoplasma*, *Ureaplasma* and other bacteria, nested PCR was performed using the primer for the first PCR (Bacterial Universal Primer for 1^st^ PCR) at the start. For the second (nested) PCR, four kinds of primers (Bacterial Universal Primer for 2^nd^ PCR, Mycoplasma Specific Primer, Ureaplasma Specific Primer, and NotMycoUrea Bacterial Universal Primer) were used. *The amplicon sizes are described, and the amplified positons on *Escherichia coli* 16S ribosomal RNA (Accession No. J01859) are shown. Mycoplasma and Ureaplasma Specific Primers do not bind to *E*. *coli* 16S rDNA. (**B**) The sequence homology between the primers (Bacterial Universal Primer for 1^st^ PCR, Bacterial Universal Primer for 2^nd^ PCR, NotMycoUrea Bacterial Universal Primer) and the target regions of *Mycoplasma*, *Ureaplasma* and other bacteria. Seven examples are shown as representative of *Mycoplasma*, *Ureaplasma* and other bacteria, respectively. The base sequence differences between the primers and the target regions are shown in red. Two of the primers (Bacterial Universal Primer for 1^st^ PCR, Bacterial Universal Primer for 2^nd^ PCR) can detect almost all kinds of bacteria including *Mycoplasma* and *Ureaplasma* species. On the other hand, the NotMycoUrea Bacterial Universal Primer can detect almost all kinds of bacteria, but does not detect *Mycoplasma* or *Ureaplasma* species because of the primer’s low sequence homology, which is a key point of our method. (**C**) The PCR amplification products of *Mycoplasma*, *Ureaplasma* and other bacteria amplified by each primer set. Six examples are used as representative of *Mycoplasma*, *Ureaplasma* and other bacteria, respectively. The gels showed no bacterial contamination using eukaryote-made thermostable DNA polymerase and also showed the specificity of each primer set. PCR amplification products were detected precisely according to the presence or absence of the targeted bacterial DNA templates.

To construct a sensitive and specific detection assay for *Mycoplasma*, *Ureaplasma* and other bacteria in amniotic fluid samples, we applied a nested PCR assay (Fig 2A) employing devised primer sets (Table 1). Using the current protocols, the limit of detection (*Escherichia coli*) of this assay is 0.74 CFU/PCR tube (37 CFU/ml of amniotic fluid). The sequence homology between the primers (Bacterial Universal Primer for 1^st^ PCR, Bacterial Universal Primer for 2^nd^ PCR, NotMycoUrea Bacterial Universal Primer) and the target regions of *Mycoplasma*, *Ureaplasma* and other bacteria are shown in Fig 2B, which indicates the strategy used for our method. For the first PCR, the Bacterial Universal Primer for 1^st^ PCR can amplify almost all kinds of bacteria including *Mycoplasma* and *Ureaplasma* species. For the second (nested) PCR, the Bacterial Universal Primer for 2^nd^ PCR can also detect almost all kinds of bacteria including *Mycoplasma* and *Ureaplasma* species. On the other hand, the NotMycoUrea Bacterial Universal Primer can detect almost all kinds of bacteria, but does not detect *Mycoplasma* and *Ureaplasma* species because of the primer’s low sequence homology, which is a key point for our method. Using these universal primers and the Mycoplasma/Ureaplasma-Specific Primers, targeted species can be correctly detected (Fig 2C), and mixed amniotic fluid infections with *Mycoplasma*, *Ureaplasma* and/or other bacteria can be clearly distinguished (Table 2). Importantly, no bacterial amplicons were observed in the negative control (distilled water) after the second (nested) PCR (also shown in Fig 2C). This would be nearly impossible without the eukaryote-made thermostable DNA polymerase.

**Table 2:** Interpretation scheme for the absence or presence of *Mycoplasma*, *Ureaplasma*, and other bacteria in amniotic fluid samples.

Bacterial Universal Primer for 2^nd^ PCR	Mycoplasma Specific Primer	Ureaplasma Specific Primer	NotMycoUrea Bacterial Universal Primer	Interpretation of absence or presence
Amplicon size (bp) 287	Amplicon size (bp) 169 or 173	Amplicon size (bp) 128	Amplicon size (bp) 120	
–	–	–	–	None
+	+	–	–	*Mycoplasma*
+	–	+	–	*Ureaplasma*
+	–	–	+	Other bacteria
+	+	+	–	*Mycoplasma* and *Ureaplasma*
+	+	–	+	*Mycoplasma* and other bacteria
+	–	+	+	*Ureaplasma* and other bacteria
+	+	+	+	*Mycoplasma* and *Ureaplasma* and other bacteria

(+) detection of the amplicon,

(−) non-detection of the amplicon, bp: base pairs

Other bacteria: bacteria other than *Mycoplasma* and *Ureaplasma*

### Comparison of the novel PCR and conventional culture results for detecting *Mycoplasma*, *Ureaplasma*, other bacteria and fungi

**Fig 3:**
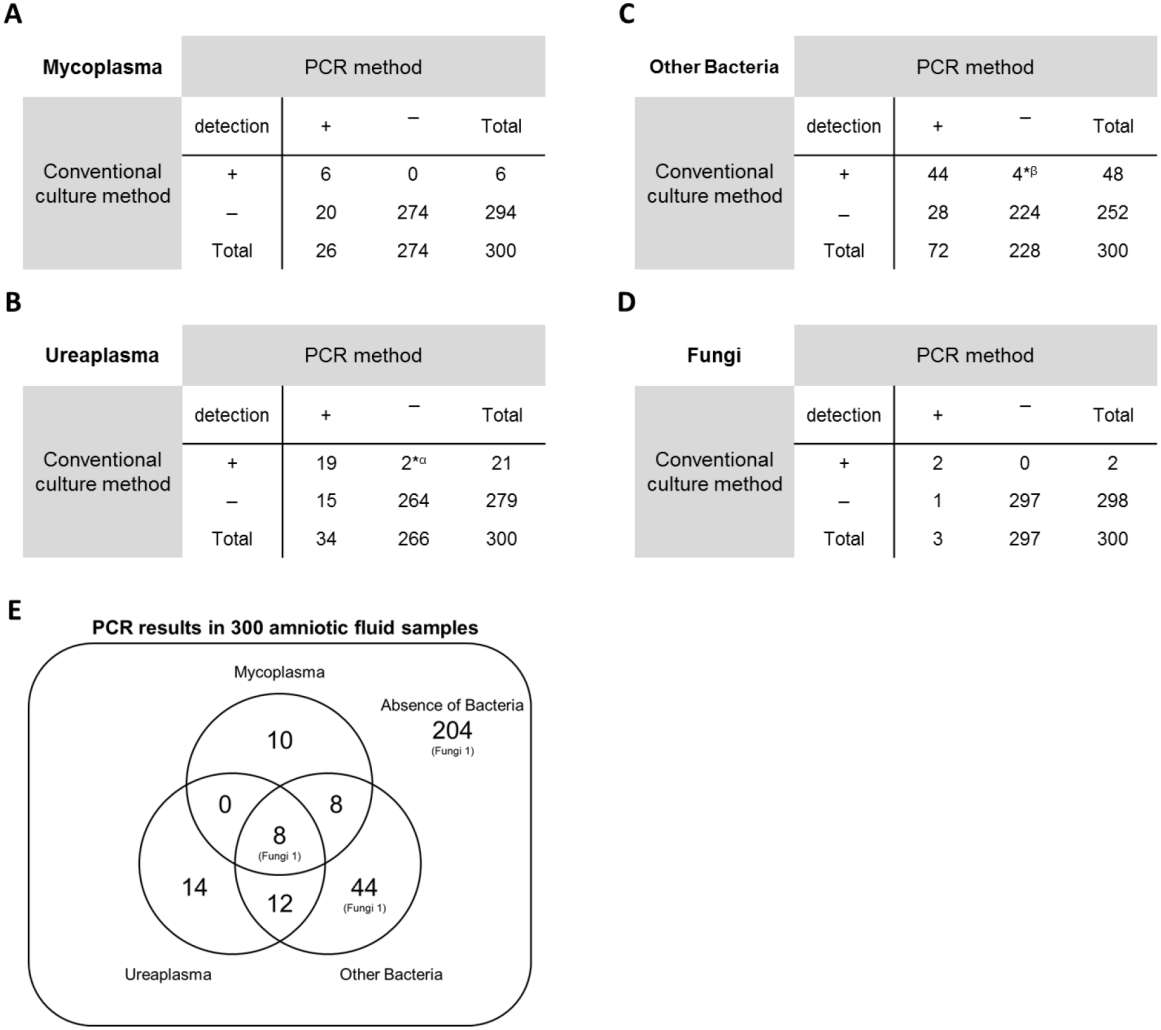
The result of a comparison of the novel PCR and conventional culture results for detecting Mycoplasma, Ureaplasma, other bacteria and fungi. Based on the detection level (positive and negative), 93.3% (280/300) of *Mycoplasma*, 94.3% (283/300) of the *Ureaplasma*, 89.3% (268/300) of other bacteria and 99.7% (299/300) of fungi results matched the culture results. (A) The numbers of cases with detected and non-detected *Mycoplasma* in 300 amniotic fluid samples. (B) The numbers of cases with detected and non-detected *Ureaplasma* in 300 amniotic fluid samples. (C) The numbers of cases with detected and non-detected bacteria other than *Mycoplasma* and *Ureaplasma* in 300 amniotic fluid samples. (D) The numbers of cases with detected and non-detected fungi in 300 amniotic fluid samples. (E) PCR results in 300 amniotic fluid samples. *α: Because of multiple colonies of other bacteria, colony-forming *Ureaplasma* could not be confirmed. In these cases, the existence of *Ureaplasma* was judged only by checking the color changes of the medium from yellow to red. *β: Four samples were PCR-negative but culture-positive, and the bacteria detected by only the culture method were *Corynebacterium species* and *Lactobacillus species* (transvaginal amniocentesis), *Corynebacterium species* and *Lactobacillus species* (vaginal delivery), *Corynebacterium species* and *Lactobacillus species* (vaginal delivery) and *Lactobacillus species* (vaginal delivery).

To assess our PCR assay, we compared the novel PCR and conventional culture results for detecting *Mycoplasma*, *Ureaplasma*, other bacteria and fungi in 300 amniotic fluid samples (Fig 3). Based on the detection level (positive and negative), 93.3% (280/300) of *Mycoplasma*, 94.3% (283/300) of *Ureaplasma*, 89.3% (268/300) of other bacteria and 99.7% (299/300) of fungi matched the culture results. For the detection of *Mycoplasma* (Fig 3A), 26 samples were positive according to the PCR method, six of which were also positive using the culture method but 20 were negative. In general, the detection rate of *Mycoplasma species* by PCR is higher than the culture method [19–23], partly because *Mycoplasma* are usually difficult to cultivate. Meanwhile, 274 samples were negative according to the PCR method, 100% (274/274) of which were also negative based on the culture method.

As for the detection of *Ureaplasma* (Fig 3B), 34 samples were positive according to the PCR method, 19 of which were also positive using the culture method, but 15 were negative. This is because *Ureaplasma species* are also usually difficult to cultivate. Meanwhile, 266 samples were negative according to the PCR method, 99.2% (264/266) of which were also negative based on the culture method. Here, two samples were PCR-negative but culture-positive. In these cases, because of multiple colonies of other bacteria, colony-forming *Ureaplasma* could not be confirmed, and the existence of *Ureaplasma* was judged only by checking the color changes of the medium from yellow to red. That is why we could not confirm the existence of *Ureaplasma* in these two samples.

With regard to the detection of bacteria other than *Mycoplasma* and *Ureaplasma* (Fig 3C), 72 samples were positive according to the PCR method, 44 of which were also positive using the culture method, but 28 were negative. In general, PCR detects more than culture, because it can also detect bacteria that are difficult to cultivate [16]. Meanwhile, 228 samples were negative according to the PCR method, 98.2% (224/228) of which were also negative based on the culture method. Because we used the eukaryote-made thermostable DNA polymerase, the nested PCR method (first PCR: 30 cycles ➞ dilute 500-fold ➞ second, nested PCR: 30 cycles) can also be applied to accurately diagnose the absence of bacteria in amniotic fluid samples. In this case, four samples were PCR-negative but culture-positive. We collected samples for the PCR method and for the culture method separately, and the bacteria detected by only the culture method were *Corynebacterium species* and *Lactobacillus species* (transvaginal amniocentesis), *Corynebacterium species* and *Lactobacillus species* (vaginal delivery), *Corynebacterium species* and *Lactobacillus species* (vaginal delivery) and *Lactobacillus species* (vaginal delivery). There is a contamination risk by normal vaginal flora when the transvaginal route is used for sampling. Therefore, we supposed that contamination might have accidentally occurred in the samples used for the culture method during sampling.

With regard to the detection of fungi (Fig 3D), three samples were positive according to the PCR method, two of which were also positive using the culture method, but one sample was negative. Meanwhile, 297 were negative according to the PCR method, 100% (297/297) of which were also negative based on the culture method.

### Comparison of the relative abundance of *Mycoplasma*, *Ureaplasma*, and/or other bacteria in amniotic fluid samples with mixed infection

To assist in the selection of antibiotics for the successful treatment of patients with PTL, it could be important to know the relative abundance of *Mycoplasma*, *Ureaplasma*, and/or other bacteria in cases of mixed infection. We therefore tried to compare the relative abundance in 28 amniotic fluid samples with mixed infection (Fig 3E, and Table 3). The mixed infections were determined by PCR, and the 28 samples were collected from women in preterm labor. In order to compare the relative abundance of *Mycoplasma*, *Ureaplasma*, and/or other bacteria in a sample, the cycle threshold (Ct) values obtained by the Mycoplasma Specific Primer, Ureaplasma Specific Primer and NotMycoUrea Bacterial Universal Primer were compared. The amplicon obtained by the Bacterial Universal Primer for 2^nd^ PCR shows only the existence of bacteria in a sample. It must be considered that the 16S ribosomal RNA (rRNA) operon copy number in genomes varies among *Mycoplasma*, *Ureaplasma*, and other bacteria from one (e.g. *Mycoplasma genitalium*) to as many as 13 copies (e.g. *Bacillus cereus*) (Table 4). This variation is a problem for comparing the relative abundance of *Mycoplasma*, *Ureaplasma*, and/or other bacteria in unknown mixed populations using 16S rRNA based approaches. To solve this problem, we tried to calculate a Ct value difference by which we could compare the relative abundance even if the difference of each 16S rRNA operon copy number was high. The amplification efficiency of our PCR method employing eukaryote-made thermostable DNA polymerase was 92% per cycle at that time (data not shown). In order to judge dominance by comparing the Ct values, at least a 3.95 cycle difference is required (13 < 1.92^Ct^). For example, for patient sample #9, the Ct value obtained by the Ureaplasma Specific Primer was 7.03, and the Ct value obtained by the NotMycoUrea Bacterial Universal Primer was 2.86. The difference between these Ct values was 4.17 cycles, which was higher than 3.95 cycles. Therefore, we judged that other bacteria were more abundant than *Ureaplasma* in this mixed infection. We compared the relative abundance of *Mycoplasma*, *Ureaplasma*, and/or other bacteria in 28 amniotic fluid samples with mixed infection in the same way as described above.

**Table 3:** The results of a comparison of the relative abundance of *Mycoplasma*, *Ureaplasma*, and/or other bacteria in amniotic fluid samples with mixed infection.

	Obtained Ct (threshold cycle) values						
Sample	Bacterial Universal Primer for 1^st^ PCR	Mycoplasma Specific Primer	Ureaplasma Specific Primer	NotMycoUrea Bacterial Universal Primer	Comparison of the relative abundance	Clinical findings	Culture-based results
**Control**							
*M*. *hominis*	11.37	11.72			*Mycoplasma*		
*U*. *parvum*	2.97		3.28		*Ureaplasma*		
*E*. *coli*	9.91			10.25	Other bacteria		
*L*. *crispatus*	4.72			5.20	Other bacteria		
**Patient**							
1	11.88	24.75		13.62	Other bacteria > *Mycoplasma*	Previous cesarean section	negative
2	13.04	26.03		13.89	Other bacteria > *Mycoplasma*	Fetal distress	negative
3	14.40	23.79		15.10	Other bacteria > *Mycoplasma*	Previous cesarean section	negative
4	14.73	24.13		15.17	Other bacteria > *Mycoplasma*	Previous cesarean section	negative
5	15.52	23.18		16.34	Other bacteria > *Mycoplasma*	Hypertension,gestational diabetes mellitus	*Mycoplasma species*
6	15.78	27.71		17.07	Other bacteria > *Mycoplasma*	Preterm labor	negative
7	22.38	27.87		22.99	Other bacteria > *Mycoplasma*	Preterm labor	*Propionibacterium acnes*
8	12.63	21.04		13.31	Other bacteria > *Mycoplasma*	Previous cesarean section	negative
9	2.56		7.03	2.86	Other bacteria > *Ureaplasma*	Premature rupture of membranes	*Lactobacillus speciesCandida albicansStreptococcus agalactiaeUreaplasma species*
10	2.78		12.00	3.34	Other bacteria > *Ureaplasma*	Dichorionic diamniotic twins	*Gardnerella vaginalisLactobacillus speciesUreaplasma species*
11	2.85		3.41	15.87	*Ureaplasma* > Other bacteria	Preterm labor	*Ureaplasma species*
12	3.07		5.49	8.00	*Ureaplasma* > Other bacteria	Premature rupture of membranes	*Prevotella biviaUreaplasma species*
13	3.48		4.47	17.95	*Ureaplasma* > Other bacteria	Preterm labor	*Ureaplasma species*
14	4.12		4.16	15.71	*Ureaplasma* > Other bacteria	Premature rupture of membranes	*Ureaplasma species*
15	6.00		21.65	8.43	Other bacteria > *Ureaplasma*	Premature rupture of membranes	*Gardnerella vaginalisStaphylococcus epidermidisStreptococcus constellatusEscherichia coliPrevotella loescheiiBacteroides fragilisUreaplasma species*
16	7.09		12.11	7.89	Other bacteria > *Ureaplasma*	Premature rupture of membranes	*Lactobacillus speciesPropionibacterium granulosumUreaplasma species*
17	7.20		17.77	8.76	Other bacteria > *Ureaplasma*	Intrauterine fetal death	*Escherichia coliStaphylococcus lugdunensisBacteroides vulgatusLactobacillus speciesBifidobacterium species*
18	12.91		13.55	14.65	*Ureaplasma* > Other bacteria	Dichorionic diamniotic twins	*Staphylococcus epidermidisEscherichia coliUreaplasma species*
19	14.04		14.9	15.35	*Ureaplasma* > Other bacteria	Formation of the bag	negative
20	14.07		22.24	14.28	Other bacteria > *Ureaplasma*	Preterm labor	negative
21	2.48	2.98	10.49	4.53	*Mycoplasma* > Other bacteria > *Ureaplasma*	Premature rupture of membranes	*Gardnerella vaginalisPeptostreptococcus magnusPeptoniphilus asaccharolyticusPrevotella biviaPeptostreptococcus anaerobiusMycoplasma speciesUreaplasma species*
22	2.61	4.44	7.47	3.63	Other bacteria > *Mycoplasma* > *Ureaplasma*	Premature rupture of membranes	*Klebsiella pneumoniaeEnterococcus faecalisMycoplasma speciesUreaplasma species*
23	3.02	11.86	3.74	6.58	*Ureaplasma* > Other bacteria > *Mycoplasma*	Premature rupture of membranes	*Gardnerella vaginalisCorynebacterium speciesStaphylococcus epidermidisLactobacillus speciesLactobacillus acidophilusMycoplasma speciesUreaplasma species*
24	6.47	24.99	13.35	8.26	Other bacteria > *Ureaplasma* > *Mycoplasma*	Formation of the bag	*Staphylococcus schleiferiGardnerella vaginalisLactobacillus species*
25	12.16	13.32	17.29	15.10	*Mycoplasma* > Other bacteria > *Ureaplasma*	Previous cesarean section	negative
26	12.71	13.35	16.53	16.96	*Mycoplasma* > *Ureaplasma* > Other bacteria	Preterm labor	negative
27	14.11	17.67	18.35	15.30	Other bacteria > *Mycoplasma* > *Ureaplasma*	Previous cesarean section	negative
28	14.27	20.23	16.52	15.81	Other bacteria > *Ureaplasma* > *Mycoplasma*	Previous cesarean section	negative

Other bacteria: bacteria other than *Mycoplasma* and *Ureaplasma*

**Table 4:** The variations of the 16S ribosomal RNA operon copy number in genomes.

Name of *Mycoplasma*, *Ureaplasma* and other bacteria	16S ribosomal RNA operon copy number*
***Bacillus cereus***	13
***Clostridium difficile***	12
***Aeromonas hydrophila***	10
***Clostridium perfringens***	10
***Enterobacter aerogenes***	8
***Enterobacter cloacae***	8
***Klebsiella pneumoniae***	8
***Bacteroides vulgatus***	7
***Escherichia coli***	7
***Streptococcus agalactiae***	7
***Bacteroides fragilis***	6
***Enterococcus faecium***	6
***Bacteroides distasonis***	5
***Staphylococcus aureus***	5
***Staphylococcus epidermidis***	5
***Staphylococcus haemolyticus***	5
***Staphylococcus lugdunensis***	5
***Enterococcus faecalis***	4
***Lactobacillus acidophilus***	4
***Lactobacillus crispatus***	4
***Paptostreptococcus magnus***	4
***Paptostreptococcus prevotii***	4
***Pseudomonas aeruginosa***	4
***Streptococcus mitis***	4
***Eubacterium lentum***	3
***Campylobacter jejuni***	3
***Propionibacterium acnes***	3
***Acinetobacter calcoaceticus***	2
***Gardnerella vaginalis***	2
***Ureaplasma parvum***	2
***Ureaplasma urealyticum***	2
***Mycoplasma hominis***	2
***Mycoplasma genitalium***	1
***Mycoplasma pneumoniae***	1

* The 16S rRNA operon copy numbers were obtained from the Gene database at NCBI (http://www.ncbi.nlm.nih.gov/gene/).

## Discussion

In this study, we examined the detection of *Mycoplasma*, *Ureaplasma*, other bacteria and fungi in 300 amniotic fluid samples in order to assess our PCR assay, not to assess the PTL. In these 300 samples, the amniotic fluid samples collected by transvaginal amniocentesis (35 cases) or vaginal delivery (16 cases) were included. These samples are not always suitable for assessing intra-amniotic infection, because of the comparatively high contamination risks by normal vaginal flora. That is why the positive infection rates of *Mycoplasma*, *Ureaplasma*, other bacteria and fungi in Fig 3 do not necessarily reflect the real infection rates in the amniotic fluid. But most of the samples (249 cases) were obtained by abdominal amniocentesis or obtained at cesarean section. These samples were less likely to be contaminated.

In order to accurately diagnose bacterial infection, we developed a bacteria-free PCR system. Because we use the eukaryote-made thermostable DNA polymerase, our PCR assay can also be applied to accurately diagnose the absence of bacteria in patient samples. It is important to rapidly distinguish bacterial causes from non-bacterial causes for the choice of antibiotics in intra-amniotic infection. Commercial thermostable DNA polymerases are known to have contamination with host-derived bacterial DNA. When using bacterial universal primers for PCR detection, the contaminating bacterial amplicons can be observed by the 40th cycle of PCR amplification [28]. We applied a nested PCR assay (first PCR: 30 cycles ➞ dilute 500-fold ➞ second, nested PCR: 30 cycles) in which, in almost all positive samples, amplification curve was observed only in the second (nested) PCR. So according to our results, more than 40 cycles of PCR amplification is usually required to detect pathogens directly from amniotic fluid samples. For this reason, without the eukaryote-made thermostable DNA polymerase, it would be nearly impossible to detect the bacterial isolate directly from an amniotic fluid sample. At present, we are working to make this eukaryote-made thermostable DNA polymerase commercially available.

With regard to the detection of bacteria other than *Mycoplasma* and *Ureaplasma* (Fig 3C), 72 samples were positive according to the PCR method, but of these 72 samples, 28 were negative using the culture method. Unlike detecting *Mycoplasma* and *Ureaplasma*, when detecting other bacteria by NotMycoUrea Bacterial Universal Primer, we always have to consider the contamination risks during the PCR procedures. The sensitivity of our method was high (37 CFU/mL), so these 28 samples did have the possibility of contamination during PCR procedures. However, to minimize the contamination risks, we set two kinds of negative control, a negative control for DNA extraction and a negative control for the PCR step. In this way, we always checked the contamination risk, so we believe these 28 samples did not have contamination.

To compare the relative abundance of *Mycoplasma*, *Ureaplasma*, and/or other bacteria more precisely in amniotic fluid samples with mixed infection, we devised three primer designs. A Mycoplasma Specific Primer, Ureaplasma Specific Primer and NotMycoUrea Bacterial Universal Primer were designed to obtain similar sizes of amplicons (120, 169 or 173, and 128 base pairs, respectively) with similar fluorescence intensity. A large difference in the amplicon size affects the Ct values to some extent. For example, the Ct values obtained by the Bacterial Universal Primer for 2^nd^ PCR were usually a little bit lower than the Ct values obtained by the other primers because the amplicon size for this is larger than that of the other amplicons. In addition, the base sequence differences between the primers and the target regions also affect the Ct values. Because of this, we are now planning to mix several kinds of primers with no mismatches, such as Mycoplasma Specific Primer (Table 1), in one tube. In this way, the precise comparison of the relative abundance of *Mycoplasma*, *Ureaplasma*, and/or other bacteria in amniotic fluid samples with mixed infection is technically not easy, but for assisting in the selection of antibiotics, it could be more helpful than the detection results alone. To establish a precise comparison of the relative abundance, further developments and refinements of the procedures and sequences will be required.

In conclusion, using the eukaryote-made thermostable DNA polymerase, we developed a novel PCR assay for the rapid detection of *Mycoplasma*, *Ureaplasma*, other bacteria and fungi in amniotic fluid samples within three hours of sample collection. Employing the devised primer sets, mixed amniotic fluid infections of *Mycoplasma*, *Ureaplasma*, and/or other bacteria can be clearly distinguished. Moreover, this PCR assay can be used to rapidly diagnose the absence of bacteria in clinical samples. We also tried to compare the relative abundance of *Mycoplasma*, *Ureaplasma*, and/or other bacteria in amniotic fluid samples with mixed infection, and showed the possibility that this technique can assist in the selection of antibiotics. We hope that this PCR assay will positively contribute to the treatment of intra-amniotic infection and the prevention of preterm delivery.
